# Tremor in multiple system atrophy: a systematic literature review

**DOI:** 10.1007/s00702-026-03129-9

**Published:** 2026-03-02

**Authors:** Maria Schneller, Frank Jagusch, Bianca Calio, Lukas Gattermeyer-Kell, Daniela Kern, Melanie Suette, Igor Kuchin, Petra Katschnig-Winter, Mariella Kögl, Uwe Siebert, Florian Krismer, Beate Jahn, Alessandra Fanciulli, Petra Schwingenschuh

**Affiliations:** 1 Department of Neurology, Medical University of Graz, Graz, Austria; 2 Department of Neurology, Medical University of Innsbruck, Innsbruck, Austria; 3 Institute of Public Health, Medical Decision Making and Health Technology Assessment, Department of Public Health, Health Services Research and Health Technology Assessment, UMIT TIROL – University for Health Sciences and Technology, Hall in Tirol, Austria; 4 Division of Health Technology Assessment and Bioinformatics, ONCOTYROL - Center for Personalized Cancer Medicine, Innsbruck, Austria; 5 Center for Health Decision Science, Departments of Epidemiology and Health Policy & Management, Harvard T.H. Chan School of Public Health, Boston, MA USA; 6 Institute for Technology Assessment, Department of Radiology, Massachusetts General Hospital, Harvard Medical School, Boston, MA USA

**Keywords:** Multiple system atrophy, Tremor, Parkinson’s disease

## Abstract

Up to 80% of patients with multiple system atrophy (MSA) exhibit tremor or tremor-like phenomena, yet the clinical features of these manifestations remain insufficiently characterized. Clearer phenotypic delineation may support differential diagnosis from other tremor-associated disorders, particularly Parkinson’s disease (PD). We aimed to determine characteristics and prevalence of tremor, distribution across MSA phenotypes and body regions, and its treatment responsiveness. We systematically searched PUBMED for articles reporting tremor in MSA, following PRISMA guidelines. Eligible studies were screened, with data reviewed by two reviewers and risk of bias assessed using JBI critical appraisal tools. Of 127 records, 25 studies met inclusion criteria, comprising 963 MSA patients. Tremor types and prevalence differed by phenotype. In MSA-P, rest tremor occurred in 26.5–38.1% and action tremor in 60.3–81.8%. In MSA-C, rest tremor occurred in 11-22.5%, action tremor in 44.9–97.5%. Among action tremor, a 3-Hz pseudo-orthostatic tremor occurred more often in MSA-C (76.3–97.5%) than MSA-P (24.1–38.3%). Polyminimyoclonus was present in 16.5% of MSA-P and 10.5% of MSA-C. Neurophysiologically, MSA-C typically showed ~ 3-Hz pseudo-orthostatic tremor, whereas MSA-P presented a 7–8-Hz postural tremor and 5-6-Hz rest tremor. Levodopa improved tremor in 11.8–92% of cases, with benefit more often in MSA-P and in action tremor. Preliminary reports suggest potential benefit of clonazepam and botulinum toxin, while a pilot high intensity focused ultrasound therapy showed no improvement. This first systematic review demonstrates that tremor in MSA is common and phenotypically diverse. Rest tremor is generally less frequent than action tremor and a 3-Hz pseudo-orthostatic tremor is characteristic of MSA-C.

## Introduction

Multiple system atrophy (MSA) is a rapidly progressive neurodegenerative disease, characterized by different combinations of parkinsonism, cerebellar ataxia, and autonomic failure. Depending on the predominant motor phenotype, a distinction between the parkinsonian (MSA-P) and the cerebellar subtype (MSA-C) is made (Wenning et al. 2022). Although MSA and Parkinson’s disease (PD) share many clinical features, recent diagnostic criteria emphasize that classical PD signs are rare in MSA, whereas jerky myoclonic postural or action tremor is considered a supportive motor feature for MSA (Wenning et al. 2022). Due to the underlying neuropathological involvement of both basal ganglia and pontocerebellar circuits, patients with MSA often exhibit multiple tremor types.

Clinical studies to date provide only limited and inconsistent data on the prevalence and clinical characteristics of tremor in MSA (Mailankody et al. 2017).

According to previous reports, tremor occurred in up to 80% of patients with MSA. Postural tremor typically dominates, while the classic resting tremor, known as “pill-rolling”, occurred in only a minority of cases. Jerky or irregular involuntary movements of the hands and fingers are commonly observed in patients with MSA during posture maintenance (Kaindlstorfer et al. 2013). However, since tremor is defined as rhythmic and oscillatory movements, these irregular movements, accurately described as “Polyminimyoclonus”, do not meet the formal criteria (Bhatia et al. 2018). Myoclonus, often occurring independently of postural tremor, is not uncommon in MSA (Mailankody et al. 2017). Because these phenomena closely mimic tremor, they directly contribute to the inconsistent clinical characterization of tremor in MSA, and the lack of standardized documentation further complicates the differentiation between MSA and PD.

In light of this diagnostic complexity, two recent developments have reshaped the conceptual and classificatory framework: the updated consensus statement on tremor classification proposed by the International Parkinson and Movement Disorder Society (MDS) (Bhatia et al. 2018) and the revised diagnostic criteria for MSA (Wenning et al. 2022), which now explicitly integrate tremor characteristics. Moreover, several new reports on tremor in MSA have appeared since the last review was published in 2013. However, there has been no systematic evaluation and synthesis of these reports to date, nor have these findings been summarized in the context of the updated classification and diagnostic standards.

Addressing this gap, the present systematic literature review aims to identify the prevalence and clinical phenomenology of tremor in patients with MSA, and treatment responsiveness by systematically analysing and synthesizing studies published since 2013 that examined tremor in clinically diagnosed MSA populations.

## Methods

A systematic literature search of German- and English-language journal articles was performed in PUBMED from January 2013 to May 2025 (See Appendix). In addition, reference tracking of relevant articles yielded nine additional publications. The literature research was conducted according to the *Preferred Reporting Items for Systematic reviews and Meta-Analyses* (PRISMA) guidelines. In order to check the relevance of the publications, both abstracts and full texts were screened using terms such as “tremor” or “MSA”. We included original experimental, descriptive (including case reports), and analytical observational studies on patients with MSA that reported tremor and provided specific information on tremor type, activation conditions, or related characteristics. We excluded all articles without original data or without any description or occurrence of tremor in MSA. Two researchers (MS and PS) independently screened all identified titles and abstracts and subsequently assessed full-text articles of potentially eligible studies, according to these inclusion and exclusion criteria. Discrepancies between them were resolved through discussion and consensus. We developed standardized evidence tables to systematically extract information including study characteristics (design, country, sample size, sample characteristics), detailed tremor description, diagnostic measures, treatment response measures, key outcomes and risk of bias. Extracted evidence was synthesized narratively and using figures due to heterogeneity in definitions and methods. In addition, we used the standardized *Joanna Briggs Institute* (JBI) critical appraisal tools for each study design (Moola et al. 2024) to address internal validity, risk of information bias, selection, confounding and design biases and the clarity of reporting (Munn et al. 2019). Each JBI item was rated as fulfilled, not fulfilled, unclear, or not applicable. We calculated the percentage of “Fulfilled” responses on the JBI checklist for each study and included studies that met the predefined selection criteria and achieved a quality assessment of 50%. Results are reported in systematic evidence tables. Pie charts are used to report geographical distributions of diagnosed MSA phenotypes. Results are reported in systematic evidence tables (See Appendix). Pie charts are used to report geographical distributions of diagnosed MSA phenotypes.

Solely for the report of tremor prevalence in MSA, we excluded studies with *n* < 40 to avoid imprecise prevalence estimates. The cut-off was determined post hoc and is based on a precision criterion. In case prevalence is *p* = 0.5, the 95% margin of error at *n* = 40 is approximately ± 15% points.

The review is structured in line with axis 1 of the proposed tremor classification (Bhatia et al. 2018). Focusing on clinical characteristics, we organize the findings according to prevalence and distribution across phenotypes, body regions, neurophysiological characteristics, and responsiveness to treatment.

**Fig. 1:**
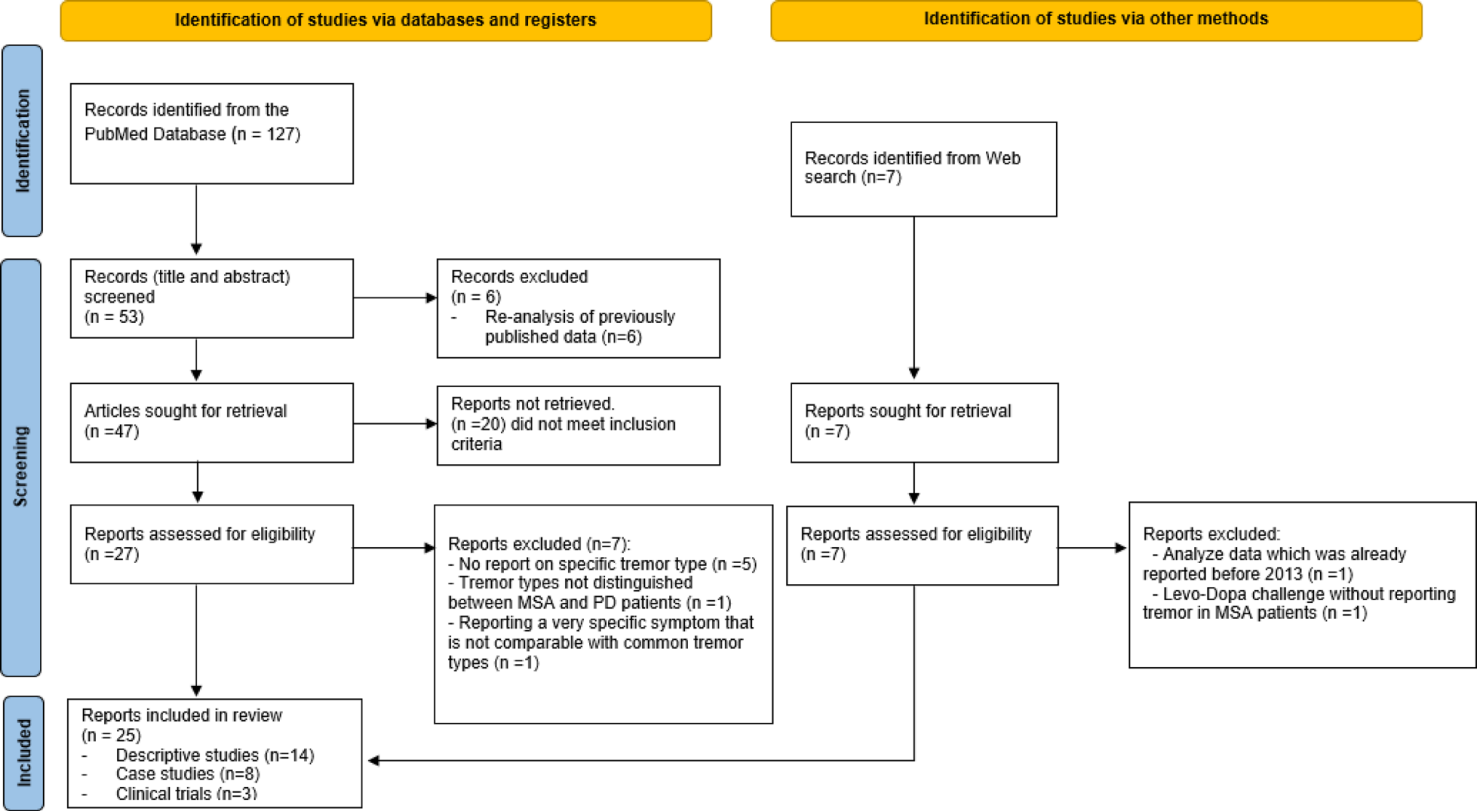
Search strategy of literature review based on the PRISMA 2020 flow diagram for systematic reviews (Page et al. 2021)

## Results

### Study characteristics

**Fig. 2:**
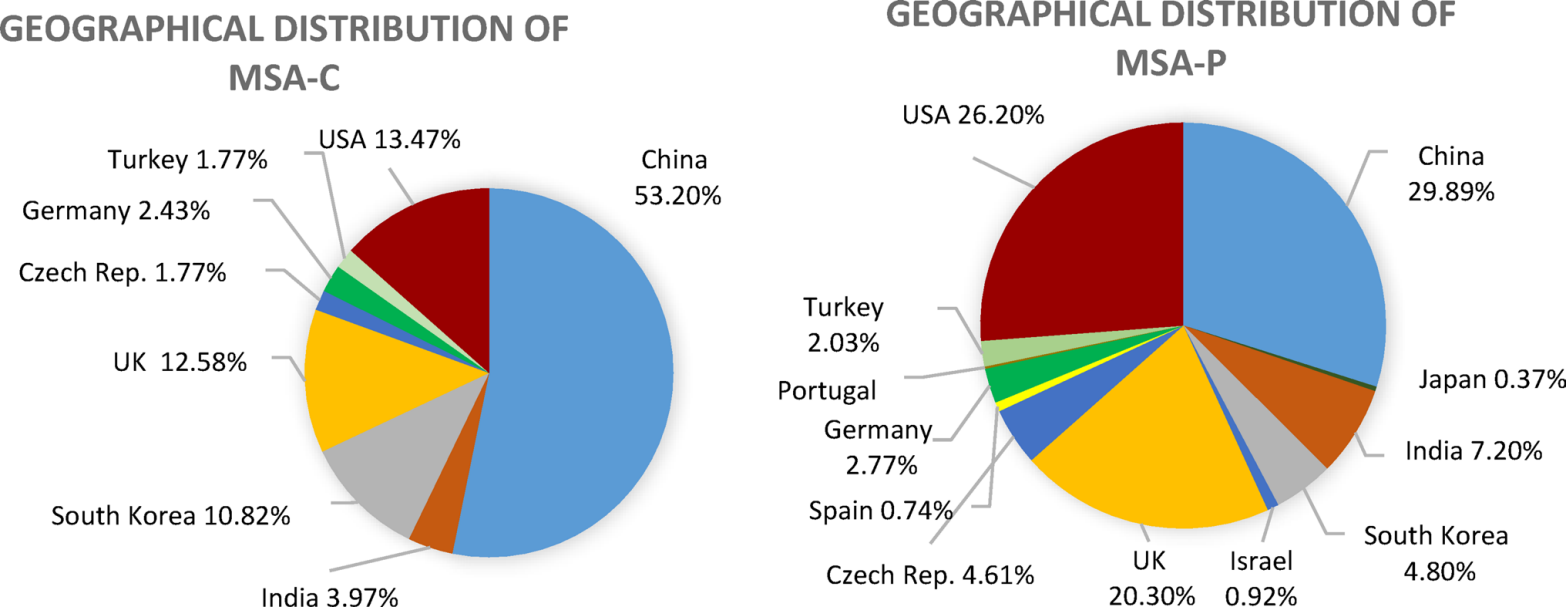
Geographical percentage distribution of diagnosed MSA phenotypes

We identified 127 records via PUBMED, whereof a total of 47 articles remained after title and abstract screening. Seven records were additionally identified through web searches. In total, twenty-five publications (20 from PUBMED search, five from web search) reporting tremor in MSA met the predefined selection criteria and met the ≥ 50% threshold of quality assessment. The mean quality across all studies was 84.62%. The selection process is illustrated in the PRISMA flowchart (Fig. 1). Among the included studies, 14 were descriptive studies, eight were case reports, and three were clinical trials. Collectively, these studies encompass 963 patients with MSA (545 male, 418 female), comprising 510 with MSA-P (53%) and 453 with MSA-C (47%). The geographic distribution spans 12 countries, with phenotype distribution across countries summarized in the pie charts in Fig. 2. In both, European and US studies, MSA-P diagnosis is more frequent than MSA-C (Europe: 166 MSA-P vs. 76 MSA-C; US: 142 MSA-P vs. 61 MSA-C), while the opposite applies for Asian studies, with MSA-C being more frequent than MSA-P (308 MSA-C vs. 234 MSA-P). However, only 18.9% of all included cases were autopsy-confirmed, encompassing 164 patients from the UK (103 MSA-P, 61 MSA-C) (Batla et al. 2013; Miki et al. 2019), 19 cases from the US (7 MSA-P, 12 MSA-C) (Xie et al. 2015) and single cases from Japan (1 MSA-P) (Fukushima et al. 2021) and Spain (1 MSA-C) (Natera-Villalba et al. 2022).

### Tremor prevalence

**Fig. 3:**
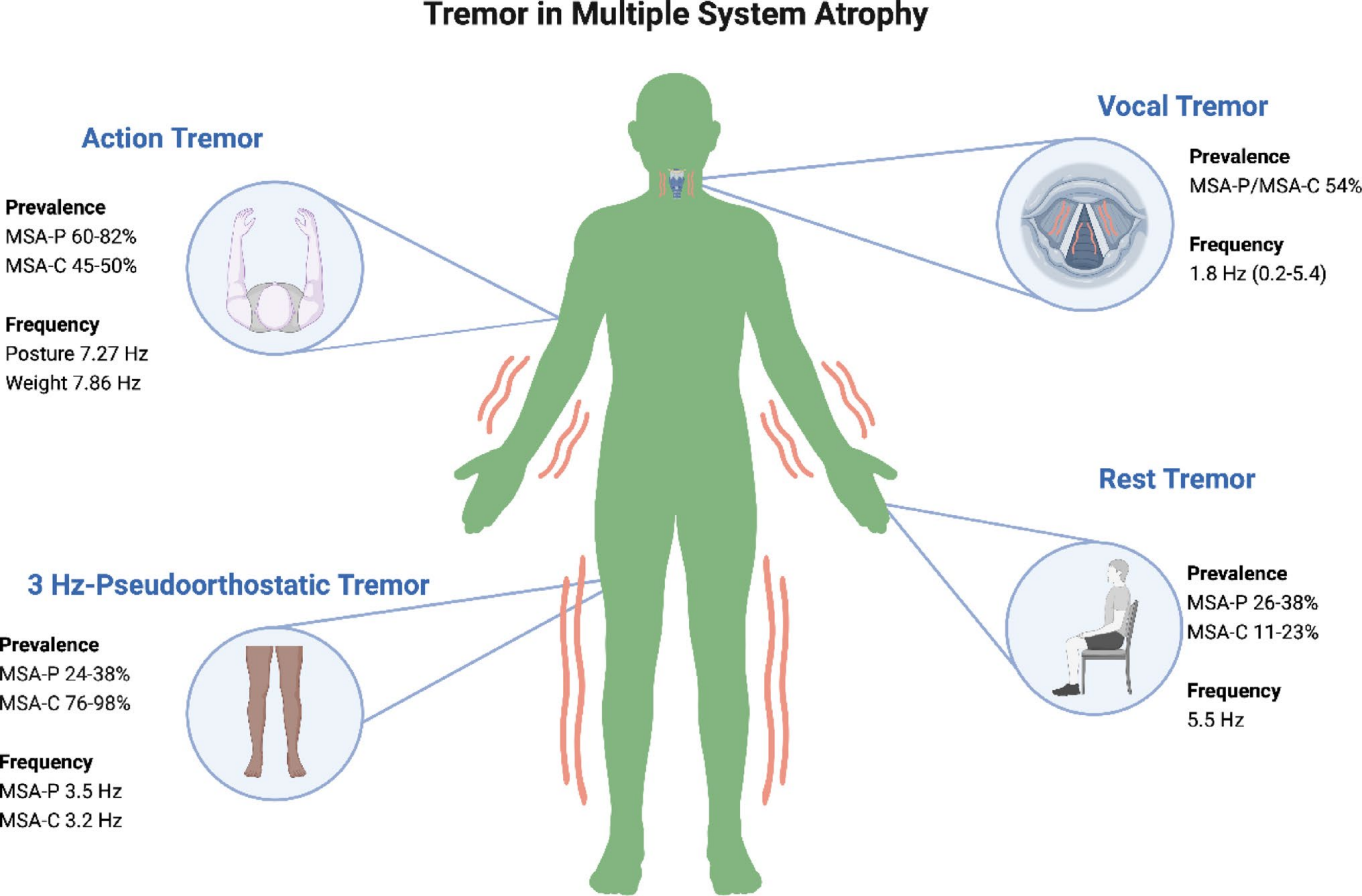
Overview of tremor in multiple system atrophy. Note: Prevalence estimates were rounded to whole numbers. Created in BioRender. Jagusch, F. (2026) https://BioRender.com/5m83t8x.

Across nine studies (Low et al. 2015; Jeong et al. 2017; Li et al. 2018, 2021; Miki et al. 2019; Zhao et al. 2020; Pradhan and Tandon 2023; Wang et al. 2025) meeting the sample size criterion (*n* > 40; See Methods), set exclusively for prevalence estimation, comprising *n* = 751 patients with MSA (365 MSA-P, 386 MSA-C), the prevalence of tremor was as follows: the overall prevalence of tremor ranged from 10.5 to 97.0%. In MSA-P, rest tremor occurred in a range from 26% to 38% (Low et al. 2015; Zhao et al. 2020; Pradhan and Tandon 2023). Action tremor prevalence occurred in 24% − 82% (Low et al. 2015; Li et al. 2018; Zhao et al. 2020; Wang et al. 2025) and included pseudo-orthostatic tremor, defined as a lower-limb tremor while standing with a frequency below 13-Hz (Erro et al. 2014), while excluding jerky, myoclonic tremor. In MSA-C, rest tremor occurred between 11% and 23% (Low et al. 2015; Jeong et al. 2017; Zhao et al. 2020; Pradhan and Tandon 2023). Action tremor occurred between 44.9 and 97% (Low et al. 2015; Li et al. 2018, 2021; Zhao et al. 2020; Wang et al. 2025), with particularly high prevalence of pseudo-orthostatic tremor. Table 1 provides the study-specific percentages of the included studies. An overview of tremor types, prevalence and affected body regions is also provided with Fig. 3.

In addition, polyminimyoclonus was reported in 16.5% of patients with MSA-P and in 10.5% of patients with MSA-C (Miki et al. 2019). Most articles did not specify the body region affected by the tremor; however, when reported, upper limb tremor was more frequently mentioned in MSA-P (Shindo et al. 2014; Brum et al. 2016; Su et al. 2020; Mir et al. 2024) and lower limb tremor more frequent in MSA-C (Li et al. 2018, 2021; Wang et al. 2025). It should also be noted that a subset of the patients (*n* = 207) was from retrospective cohorts, and the diagnosis has only been autopsy-confirmed in 112 patients with MSA-P and 70 with MSA-C.

**Table 1:** Prevalence of Rest and Action Tremor in MSA phenotypes

	MSA Phenotype					
	MSA-*P*			MSA-C		
Study (n)	Rest tremor %	Action tremor %	*n*	Rest tremor %	Action tremor %	*n*
Low et al. 2015 (175)	38.1	60.3	126	22.5	44.9	49
Zhao et al. 2020 (50)	27.3	81.8	22	14.3	50.0	28
Pradhan and Tandon 2023 (57)	25.6	–	39	11.1	–	18
Li et al. 2018 (69)	–	24.1	29	–	97.5	40
Li et al. 2021 (58)	–	–	–	–	82.8	58
Wang et al. 2025 (133)	–	38.3	46	–	76.3	87

Note. Dashes indicate that the respective tremor type was not reported. Sample size (n) in the first column indicates the number of participants diagnosed with MSA in this study. Action tremor includes pseudo-orthostatic tremor (lower-limb tremor while standing, frequency < 13 Hz) and excludes jerky or myoclonic tremor

### Tremor characteristics

#### Rest tremor

Rest tremor was noted across multiple studies with prevalence rates ranging from isolated cases up to 38.1% of patients with MSA (Low et al. 2015). Among the patients with reported rest tremor included in this review, 50% were classified as MSA-P, 18.7% as MSA-C, and in 31.3%, phenotype was not reported. The classic pill-rolling type was observed in 10 out of 39 patients with MSA-P and two out of 18 with MSA-C in a single study (Pradhan and Tandon 2023), and in six patients (3.8%) with unspecified phenotype (Miki et al. 2019). Other rest tremor forms were reported more often, though usually without specification, including tremor in the limbs, suprahyoid muscles and tongue (Bhattacharjee et al. 2024), and “Other” (Xie et al. 2015; Low et al. 2015; Zhao et al. 2020; Fukushima et al. 2021).

#### Action tremor

Action tremor is markedly more prevalent than rest tremor, reported in a total of 513 patients with MSA (53.3%). Among the reports where activation condition is differentiated between phenotypes, 243 individuals were diagnosed with MSA-P and 233 with MSA-C. Postural tremor is the most common manifestation. A high prevalence was observed in both subtypes, particularly in the lower limbs among patients with MSA-C (Li et al. 2018; Wang et al. 2025). Patients with MSA-P also exhibited postural tremor, but more frequently in the upper limbs (Low et al. 2015; Zhao et al. 2020). Additional reports included kinetic tremor (Mir et al. 2024) and intention tremor (Hwang et al. 2019; Miki et al. 2019). Vocal tremor is reported in three studies, comprising 30 patients with speech symptoms (Rusz et al. 2015; Hlavnička et al. 2020; Mir et al. 2024). No re-emergent tremor was reported.

#### Myoclonus

Myoclonic symptoms were reported in four different case reports of patients with MSA-P, presenting as orthostatic myoclonus (Gasca-Salas et al. 2013) or myoclonic jerks of other body regions (Batla et al. 2013; Shindo et al. 2014; Hwang et al. 2019). A larger clinicopathological study shows a prevalence of polyminimyoclonus in about 10% in MSA-C and 17% in MSA-P (Miki et al. 2019).

#### Differences between MSA-P and MSA-C

Based on the reported cases, tremor syndromes differ between MSA-P and MSA-C phenotypes (see Table 2). Rest tremor prevalence is higher in MSA-P (range: 25.6% − 38.1%) (Low et al. 2015; Zhao et al. 2020; Pradhan and Tandon 2023) compared to MSA-C (range: 11.1–22.5%) (Low et al. 2015; Jeong et al. 2017; Zhao et al. 2020; Pradhan and Tandon 2023). One article reports classic pill-rolling tremor, with a prevalence of 25.6% in MSA-P and 11.1% of MSA-C cases (Pradhan and Tandon 2023), whereas the other article to not specify the rest tremor type. Action tremor in the lower limbs while standing (3-Hz pseudo-orthostatic tremor) is more characteristic for MSA-C (Lower limbs range: 76.3–97%) than for MSA-P (Lower limbs range: 24.1–38.3%) (Li et al. 2018, 2021). Action tremor activated by posture in the upper limbs shows a wider range of occurrence frequency in MSA-P (Upper limbs range: 24.1–82%), whereas this tremor occurred in around half of patients with MSA-C (Upper limbs range: 44.9–50%). For a graphical overview, see Fig. 3. Intention tremor is only reported in one MSA-C case and 30 cases with no reference to phenotype (Hwang et al. 2019; Miki et al. 2019).

### Neurophysiological characteristics

Neurophysiological tremor analysis was performed in twelve articles (Batla et al. 2013; Gasca-Salas et al. 2013; Rusz et al. 2015; Li et al. 2018, 2021; Hwang et al. 2019; Hlavnička et al. 2020; Su et al. 2020; Calikusu et al. 2023; Mir et al. 2024; Bhattacharjee et al. 2024; Wang et al. 2025). Few studies reported detailed electrophysiological findings (summarized in Fig. 3), whereas several merely report EMG use, without detailed results.

Large-scale studies demonstrated distinct tremor characteristics in MSA subtypes, with postural lower-limb tremor around 3-Hz highly prevalent in MSA-C, affecting 76.3–97%, but present in only about 25% of patients with MSA-P. Postural lower-limb tremor showed a slow frequency in both subtypes (median 3.2-Hz, range: 2.3–4.6-Hz), with slightly higher frequency in MSA-P (3.5-Hz) than in MSA-C (3.2-Hz), whereas amplitude was greater in MSA-C (Wang et al. 2025). Upper-limb tremor frequencies in MSA-P reached 5.5-Hz at rest, 7.3-Hz in postural position, and 7.9-Hz during weight-holding (Su et al. 2020). In MSA, upper-limb tremor had a significantly higher dominant frequency across resting, posture and weight-holding conditions than in PD (Resting: 5.50 vs. 4.76-Hz; Postural: 7.27 vs. 5.60-Hz; 1000 g weight: 7.86 vs. 6.22-Hz), but this difference did not occur for the lower limbs. Dominant frequencies did not differ between MSA-P and MSA-C.

Several studies characterized vocal tremor using acoustic and electrophysiological methods. Acoustic speech analysis, measuring tremulous phonation, revealed vocal tremor in 54% of patients with MSA, with a mean frequency tremor intensity index of 1.8-Hz (range 0.2–5.4), which was significantly higher than in progressive supranuclear palsy (PSP) and patients with PD (Rusz et al. 2015). Frequency analysis identified tremor components of < 4-Hz, 4–7-Hz, and > 7-Hz, with the 4–7-Hz band showing the strongest association (Hlavnička et al. 2020). Detailed case studies described vocal flutter with 6–9 fundamental pitch fluctuations per 500 ms, pitch ranges of 121–154-Hz, jitter of 0.5–1%, and shimmer of 15–20% (Mir et al. 2024). In a single case, a 5-Hz rhythmic tremor of the anterior digastric muscles was recorded during suprahyoid rest, showing regular, non-distractible discharges (Bhattacharjee et al. 2024). Neurophysiological documentation of myoclonus is provided in a single study. In three patients with MSA-P with orthostatic myoclonus, short EMG bursts (20–97 ms) occurred without muscular co-contraction. One patient showed upper-limb reflex myoclonus with delayed somatosensory evoked potentials, whereas the others had normal latencies (Gasca-Salas et al. 2013).

**Table 2:** Tremor subtypes reported in studies, classified according to MSA phenotypes

Tremor subtype	MSA-*P*	MSA-C	Phenotype not specified
**Rest**	**Classic pill-rolling** (Pradhan and Tandon 2023) **Other** (Xie et al. 2015; Low et al. 2015; Brum et al. 2016; Su et al. 2020; Zhao et al. 2020; Fukushima et al. 2021; Bhattacharjee et al. 2024)	**Classic pill-rolling** (Pradhan and Tandon 2023) **Other** (Xie et al. 2015; Low et al. 2015; Jeong et al. 2017; Hwang et al. 2019; Zhao et al. 2020)	**Classic pill-rolling** (Miki et al. 2019) **Other** (Calikusu et al. 2023; Ye et al. 2024)
**Action**	**Postural** (Batla et al. 2013; Xie et al. 2015; Low et al. 2015; Su et al. 2020; Zhao et al. 2020; Balint et al. 2021; Fukushima et al. 2021; Natera-Villalba et al. 2022) **3-Hz Pseudo-Orthostatic** (Li et al. 2018; Wang et al. 2025) **Kinetic** (Mir et al. 2024)	**Postural** (Low et al. 2015; Zhao et al. 2020) **3-Hz Pseudo-Orthostatic** (Li et al. 2018, 2021; Wang et al. 2025) **Intention** (Hwang et al. 2019)	**Postural** (Miki et al. 2019) **Intention** (Miki et al. 2019)
**Vocal**	“**Vocal flutter**” (Mir et al. 2024) **Vocal tremor** (Rusz et al. 2015)	**Vocal tremor** (Rusz et al. 2015)	**Vocal tremor** (Hlavnička et al. 2020)
**Myoclonus**	**Orthostatic** (Gasca-Salas et al. 2013) **“Recurrent asynchronous and arrhythmic myoclonus”** (Shindo et al. 2014) **Polyminimyoclonus** (Miki et al. 2019)	**Polyminimyoclonus** (Miki et al. 2019)	–

### Response to treatment

Fourteen studies report treatment responsiveness of tremor (Shindo et al. 2014; Xie et al. 2015; Low et al. 2015; Brum et al. 2016; Jeong et al. 2017; Miki et al. 2019; Zhao et al. 2020; Balint et al. 2021; Fukushima et al. 2021; Natera-Villalba et al. 2022; Pradhan and Tandon 2023; Mir et al. 2024; Bhattacharjee et al. 2024; Ye et al. 2024). A levodopa challenge test demonstrated a lack of responsiveness in individuals with atypical Parkinsonism. MSA-P was more common among the group of patients benefiting from levodopa, compared to MSA-C. Observational studies revealed heterogeneous but often higher rates of initial responsiveness, with 56%-92.3% in MSA-P, 25%-72.2% in MSA-C (Low et al. 2015; Pradhan and Tandon 2023) and sustained benefit in 64% (Pradhan and Tandon 2023). While two articles report 40.5–45.5% responders (Jeong et al. 2017; Miki et al. 2019), one of which reported a daily given levodopa dose of 586.7 mg (Miki et al. 2019), others found good initial response in only 24%, which did not persist beyond one year (Zhao et al. 2020). Benefit between two to ten years was reported only in sporadic cases, treated with 200–1300 mg/day levodopa (Xie et al. 2015). Rest tremor improved in about 20%, and action tremor in 37% (Ye et al. 2024).

In case reports, evidence ranged from no response (Fukushima et al. 2021; Natera-Villalba et al. 2022) to mild or partial improvement (Shindo et al. 2014; Brum et al. 2016; Balint et al. 2021; Bhattacharjee et al. 2024). Short-term improvement with levodopa in three patients with vocal tremor either diminished within six months or was complicated by adverse effects (Mir et al. 2024).

In patients with orthostatic myoclonus, piracetam showed a mild benefit, but clonazepam was ineffective (Gasca-Salas et al. 2013). Combination therapy (clonazepam, valproic acid, gabapentin, levetiracetam, trihexyphenidyl, levodopa, and baclofen) markedly improved jerky limb myoclonus in a single case, though opsoclonus persisted mildly (Shindo et al. 2014). In Sinai et al. (Sinai et al. 2024), high-intensity magnetic resonance guided focused ultrasound ventral intermediate nucleus-thalamotomy in five MSA-P patients reduced median hemi-body Clinical Rating Scale for Tremor scores from 13 at baseline (range 5–17) to 0 immediately post-treatment. Tremor improvement persisted at 1 month (*n* = 5, median = 0, range 0–14), 6 months (*n* = 4, median = 3.5, range 0–10) and 12 months (*n* = 3, median = 1, range 1–4). Adverse events included transient gait ataxia, lip paresthesia, and asthenia, all resolving within 1–12 months. Botulinum toxin injections were reported in three cases (Brum et al. 2016; Balint et al. 2021; Bhattacharjee et al. 2024), with only (Bhattacharjee et al. 2024) reporting that onabotulinumtoxin improved suprahyoid tremor.

## Discussion

Drawing on evidence from case reports, a medication treatment trial, clinicopathological, neurophysiological, and imaging studies, this systematic review of 25 articles demonstrates that tremor is present in up to 97.5% of patients with MSA, with notable differences depending on phenotype and tremor type.

Our results align broadly with earlier reports (Tison et al. 2002; Kaindlstorfer et al. 2013; Mailankody et al. 2017). However, we identified a much wider prevalence range, with overall tremor prevalence ranging from 10% to 97.5%. Some studies even reported a prevalence of action/postural tremor in MSA that exceeded those of earlier cohorts of patients with PD, in whom tremor occurred in approximately 75% of patients (Koller et al. 1989; Gironell et al. 2018).

Our updated synthesis indicates a prevalence of rest tremor in approximately one in three to four patients with MSA-P (~ 26–38%), which is higher than observed in patients with MSA-C (~ 11–23%), and lower than previously reported (Kaindlstorfer et al. 2013). Classic pill-rolling tremor was only reported in two articles (Miki et al. 2019; Pradhan and Tandon 2023), with higher prevalence than reported earlier by Wenning et al. (Wenning et al. 1994). However, although isolated cases of pill-rolling rest tremor have been reported in patients with autopsy-confirmed MSA (Miki et al. 2019), the reports by Pradhan and Tandon rely solely on clinical diagnosis. In both studies, the identification of pill-rolling tremor was based on clinical observation by a single neurologist, without interobserver validation or neurophysiological confirmation. Overall, rest tremor in MSA occurred significantly less frequently than in PD, where half of patients experience rest tremor as initial disease manifestation (Kipfer 2011). Rest tremor does not reliably distinguish MSA from PSP (6–44%) or corticobasal syndrome (CBS: 19–21%), due to overlapping prevalence data (Mailankody et al. 2017).

Consistent with prior findings (Kaindlstorfer et al. 2013; Mailankody et al. 2017), we observed that postural tremor is the most common tremor type in MSA. However, while previous findings reported a predominance of postural tremor in MSA-P compared to MSA-C (Yabe et al. 2006; Wenning et al. 2013; Kaindlstorfer et al. 2013; Mailankody et al. 2017), when taking lower limb postural tremor into account, our results contradictorily suggest that on average, postural tremor with or without jerky component, has a higher occurrence in MSA-C.

Intention tremor is previously reported in less than one-third of patients with MSA-P and roughly one-third to 45% of patients with MSA-C (Kaindlstorfer et al. 2013). However, other studies rarely reported intention tremor, and when mentioned, the specific MSA subtype was not identified (Miki et al. 2019).

Importantly, re-emergent tremor, commonly observed in 4–66% of patients with PD (Mailankody et al. 2017), appears to be absent in MSA, according to a previous study and our current synthesis of evidence (Kaindlstorfer et al. 2013).

Notably, orthostatic tremor and orthostatic myoclonus, which have not been reported at all in earlier reviews, gained recent evidence in our review, as low-frequency (3-Hz) pseudo-orthostatic tremor occurred in up to 38.3% of patients with MSA-P and up to 95.7% of patients with MSA-C (Wang et al. 2025). Similarly, orthostatic myoclonus at 3-Hz was identified in three MSA-P cases (Gasca-Salas et al. 2013). While tremor of the legs, head, chin, lips, and tongue has previously been described as uncommon in MSA (Kaindlstorfer et al. 2013), our synthesis showed that vocal tremor was reported with a prevalence of 54% (Rusz et al. 2015), more common in MSA-P but also in patients with MSA-C (Rusz et al. 2015; Hlavnička et al. 2020; Mir et al. 2024).

Neuroimaging evidence (Gallea et al. 2016) substantiates these findings by demonstrating that patients with orthostatic tremor showed greater atrophy in cerebellar lobule VI, particularly lower grey matter volumes, correlating with more severe postural instability. This supports the hypothesis that cerebellar degeneration contributes to the development of orthostatic tremor, and may explain its higher prevalence in MSA-C compared to MSA-P. In line with this, Wang et al. (Wang et al. 2025) found that the 3-Hz pseudo-orthostatic tremor is not only frequent in MSA-C but absent in a large cohort of patients with PD. This suggests a potential diagnostic utility of low-frequency pseudo-orthostatic tremor as a clinical marker for distinguishing MSA from PD.

Neurophysiological data indicate that a slow 3-Hz pseudo-orthostatic tremor is characteristic of MSA-C, whereas MSA-P more frequently exhibits a higher-frequency tremor or irregular myoclonic bursts, with only mild or absent 3-Hz tremor. The 3-Hz pseudo-orthostatic tremor observed in MSA-C lies well below the frequency range of classic primary orthostatic tremor (~ 13–18-Hz) and even below that of typical pseudo-orthostatic tremor (~ 6–7-Hz) (Thomas et al. 2007). When tremor occurred in MSA-P, it tended to be faster than in typical PD: resting tremor averaged approximately 5.5-Hz, and postural or weight-holding tremor reached up to 7-Hz, exceeding PD frequencies across all activation states (Su et al. 2020). While the limbs, especially the upper limbs, were the most frequently affected body part in both MSA and PSP tremor, occasionally extending to the lower limbs and face (Fujioka et al. 2016), pseudo-orthostatic tremor has not been convincingly documented in other atypical parkinsonian syndromes. For example, in PSP only isolated cases of primary orthostatic tremor have been reported (Maréchal et al. 2024). In Corticobasal degeneration, where postural tremor is the most prevalent tremor type, the frequency is generally higher (6–8-Hz) and tremor appears more jerky and irregular than that seen in PD (Mahapatra et al. 2004), however, orthostatic tremor has not been reported. These observations indicate that a low-frequency pseudo-orthostatic tremor of the lower limbs may serve as an important differential diagnostic feature. Accordingly, objective characterization with neurophysiological investigations are crucial. This allows for instance the detection of pure postural tremor, indicating atypical parkinsonism, which appears immediately on assuming posture with a lower amplitude, higher frequency, and a broader spectral peak, compared to PD re-emergent tremor, and often displays a jerky, irregular pattern with short (< 50 ms), synchronous bursts (Schwingenschuh et al. 2025).

Given the importance of treatment response in differentiating PD from atypical parkinsonian syndromes, we synthesized the available literature on tremor response to levodopa and other treatments. According to current MSA diagnostic criteria (Wenning et al. 2022), clinically established MSA is characterized by poor levodopa responsiveness (< 30% improvement on the MDS-sponsored revision of the Unified Parkinson’s Disease Rating Scale Part III with up to 1000 mg levodopa). We found heterogeneous and generally modest tremor responsiveness to levodopa in MSA. Natural history studies reported levodopa response in 42.5%–56.7% of patients with MSA-P and 12.9%–25% with MSA-C, with average response duration of 3.3–3.5 years and 2.6–3.3 years, respectively (Wenning et al. 2013; Low et al. 2015). In line with previous studies (Wenning et al. 2013), we observed that patients with MSA-P are more likely to benefit from levodopa than MSA-C. Non-levodopa interventions, such as botulinum toxin, benzodiazepines, piracetam, targeted combinations and focused ultrasound thalamotomy have been recently explored, suggesting potential amelioration of specific tremor phenotypes in individual cases, but high-quality efficacy evidence is still lacking (Brum et al. 2016; Balint et al. 2021; Sinai et al. 2024; Bhattacharjee et al. 2024). In single cases, clonazepam lead to mild improvement in myoclonus (Shindo et al. 2014; Hwang et al. 2019), again without high-quality data. We could not confirm previous findings on levodopa-responsiveness in low-frequency orthostatic tremor (Thomas et al. 2007).

Although no deep brain stimulation (DBS) study met inclusion criteria, external case series and retrospective analyses describe only transient or limited motor benefits with neurobehavioral complications (Artusi et al. 2022; Badihian et al. 2022), arguing against routine DBS in atypical Parkinson’s syndromes.

Compared to patients with PD, patients with MSA showed significantly less motor response to levodopa, while PSP and MSA did not differ in levodopa responsiveness (Ye et al. 2024). As previously pointed out (Kaindlstorfer et al. 2013), the results of randomized controlled trials on treatment responses in MSA tremor are inconsistent and only limitedly proven.

Beyond treatment responsiveness, MSA phenotypes also differ in terms of their geographical occurrence, with MSA-P diagnoses occurring more often in European and US studies and MSA-C in Asian studies, which aligns with earlier findings (Yabe et al. 2006; Wenning et al. 2013). More specifically, we found that Asian studies observed postural tremor with higher frequency in MSA-C than in MSA-P (Li et al. 2018, 2021), whereas US (Low et al. 2015) and European studies (Miki et al. 2019), found the opposite pattern. These findings are consistent with previous ones (Mailankody et al. 2017).

Since the updated diagnostic criteria for MSA (Wenning et al. 2022) require at least one indicative marker in addition to the central clinical features, the diagnostic relevance of specific tremor phenotypes in differentiating MSA from other parkinsonian syndromes has increased. Therefore, the systematic and precise documentation of tremor in patients with MSA is essential. The International Parkinson and Movement Disorder Society’s two-axis tremor classification (Bhatia et al. 2018) offers a practical framework for this purpose, which also helps create well-defined patient subgroups that can be reassigned if the clinical picture changes (Bhatia et al. 2018). Accordingly, tremor phenotypes that have been proposed as supportive of MSA should be systematically sought and characterized electrophysiologically in future cohorts, as their presence can significantly improve diagnostic specificity in both clinical practice and research. For instance, tremor and myoclonus can be distinguished based on clinical neurophysiological investigation of agonist-antagonist contraction pattern and rhythm regularity (Schwingenschuh et al. 2025). However, an important knowledge gap concerns tremor severity and its clinical relevance in MSA, including how often tremor is the presenting symptom or tremor treatment is requested by patients. Across included studies, tremor severity was seldom quantified and validated tremor-specific scales were almost never used, with the exception of a pilot interventional case series (Sinai et al. 2024). Most reports relied on global motor scores without tremor subscores (Su et al. 2020; Ye et al. 2024), precluding inferences about tremor burden. Likewise, tremor as the initial complaint was only noted in isolated MSA-P cases, and no study systematically assessed whether tremor severity or functional impact drove treatment seeking. This highlights the need for standardized tremor-specific severity and impact assessments in future cohorts.

Such precise characterization is particularly relevant given the growing emphasis on early and accurate diagnosis to inform patient-centered care. Recognizing this, the ongoing *MeDeMSA Care* study (Fanciulli et al. 2025) aims not only to alleviate symptomatic burden and attenuate quality-of-life decline but also to tailor management strategies according to individual healthcare preferences in patients with MSA.

### Strengths and limitations

This review, which is the first that systematically analysed tremor reports in MSA, is characterized by its comprehensive methodology, including diverse study types, ranging from experimental to observational designs and a systematic quality assessment, therefore providing a broad and integrative overview of the current evidence base.

However, as all systematic reviews, our study has several limitations. First, because multiple system atrophy is a rare disease, only a limited number of larger cohort studies were available, constraining the overall evidence base. In rare diseases, smaller studies or studies reporting null or atypical findings may be less likely to be published. This selective publication may bias the available evidence base and lead to distorted prevalence estimates.

Second, the included studies were heterogeneous in study design patient populations, diagnostic criteria and outcome measures, and often lacked standardized, comparable assessments. This limited cross-study comparability and led us to present a narrative summary rather than conduct a quantitative meta-analysis.

Third, only 112 (18.9%) of included MSA patients had an autopsy-confirmed diagnosis, such that the reliance on clinical diagnostic criteria may have led to misclassification, while the uneven distribution of autopsy-confirmed cases across studies, and the lack of stratification by MSA phenotype and tremor types prevented meaningful comparative subanalysis of these cases.

Fourth, when synthesizing prevalence data, we applied a minimum sample size threshold of *n* < 40 patients with MSA to reduce the risk of imprecise prevalence estimates. This criterion was not applied to other domains addressed in this review, where smaller and exploratory studies were considered informative and therefore included. Consequently, some smaller prevalence studies were excluded, which may have affected the completeness of prevalence estimates and should be considered when interpreting these results.

Finally, methodological limitations must be acknowledged. The literature search was conducted primarily in PubMed, supplemented by reference tracking and targeted web searches. While this strategy likely captured most clinically relevant studies, eligible publications indexed exclusively in other databases may have been missed. Moreover, restricting the search to German- and English-language publications, introduces a potential language bias. Limiting inclusion to studies published from 2013 onward may also have excluded earlier relevant work, although this decision was made to ensure alignment with contemporary diagnostic criteria and assessment methods.

### Conclusion

In summary, tremor is a common but heterogeneous feature of MSA. Action tremor predominates and occurs in a high proportion of patients with MSA-C in the lower limbs and in a high proportion of MSA-P in the upper limbs, while rest tremor is less frequent. For differential diagnosis, low-frequency pseudo-orthostatic tremor and orthostatic myoclonus are particularly relevant, as they are common in patients with MSA-C but rare in patients with PD and other atypical parkinsonian syndromes. Levodopa response is generally modest in MSA compared to PD, and evidence for other treatment remains limited and of low quality. Overall, there is a notable lack of systematic phenotyping of tremor and electrophysiological characterization in MSA. Broader adoption of the MDS two-axis tremor framework and routine, standardized documentation of tremor phenomenology may facilitate earlier early detection, improve diagnostic precision, and support more targeted research.

## Data Availability

No new data were created or analysed in this study. Data sharing is not applicable to this article.

## Appendix

PUBMED Search term:

("multiple system atrophy"[MeSH Terms] OR "multiple system atrophy"[All Fields]) AND ("Parkinson Disease"[MeSH Terms] OR "Parkinsonism"[MeSH Terms] OR "Parkinson's disease"[All Fields] OR "parkinsonism"[All Fields]) AND ("tremor"[MeSH Terms] OR tremor[All Fields] OR "rest tremor"[All Fields] OR "postural tremor"[All Fields] OR "action tremor"[All Fields] OR "intention tremor"[All Fields] OR "myoclonus"[MeSH Terms] OR myoclonus[All Fields] OR "jerky tremor"[All Fields]) AND ("clinical"[All Fields] OR "clinicopathological"[All Fields] OR "imaging"[All Fields] OR "neurophysiology"[All Fields]

**Table:**

Reference; Country	JBI score	Study design	Sample size & phenotype	Years of age mean (range)	Gender	Diagnostic measures	Reports of tremor and tremor-like phenomena	Treatment response: (i) Medication / daily dose in mg; (ii) Any initial response; (iii) sustained response > 1 year
Case reports / small case series								
(Bhattacharjee et al. 2024); UK	75%	Case report	1 MSA-P	61 (N/A)	1 F	EMG, MRI	Suprahyoid tremor rest tremor	i) L-Dopa / NR ii) Yes (partial/mild) iii) NR
(Balint et al. 2021); UK	100%	Case series	2 MSA-P	NR (67–68)	2 F	Brain CT, DAT scan, Genetics, Onconeural antibodies	Upper extremity rest tremor	i) L-Dopa / NR ii) Yes (partial/mild) iii) NR
(Mir et al. 2024); USA	60%	Case series	3 MSA-P	NR (62–72)	2 M, 1 F	Acoustic electrophysiology	Vocal flutter	i) L-Dopa / 1600 or 450 QID ii) Yes iii) No
(Batla et al. 2013); UK	70%	Case series	4 MSA-P	NR (51–76)	1 M, 3 F	MRI, DAT Scan, EMG	Postural tremor, jerky tremor	i) L-Dopa / NR ii) No iii) No
(Fukushima et al. 2021); Japan	100%	Case report	1 MSA-P	81 (N/A)	1 F	MRI, DAT Scan, Autopsy	Right upper extremity rest and action tremor	i) L-Dopa up to 300 mg/day; pramipexole 0.375 mg/day ii) No (L-Dopa); Yes (pramipexole, slight) iii) Sustained response: NR
(Brum et al. 2016); Portugal	100%	Case report	1MSA-P	80 (N/A)	1 M	MRI	Upper limb rest tremor	i) L-Dopa; carbidopa / 300 ii) No iii) No
(Shindo et al. 2014); Japan	75%	Case report	1 MSA-P	48 (N/A)	1 F	MRI, SPECT	Upper extremity tremor, myoclonus	i) Polypharmacy / NR ii) Yes (mild) iii) NR
(Natera-Villalba et al. 2022); Spain	100%	Case report	1 MSA-P	67 (N/A)	1 F	MRI, DAT SPECT, Autopsy	Myoclonic postural tremor	i) NR ii) No iii) No
(Hwang et al. 2019); USA	50%	Case–control study	6 MSA-C	54.5 AO; 62.83 AD (NR)	1 M, 5 F	EMG, Patients reports, neuropathological examination of brain and spinal cord	Upper extremity rest tremor and intention tremor, myoclonus	i) Clonazepam / NR ii) Only in 1 patient iii) NR
Cross-sectional / cohort / observational studies								
(Su et al. 2020); China	100%	Descriptive cross-sectional study	93 (65 MSA-P & 28 MSA-C	60 (NR)	45 M, 48 F	UPDRS-III, EMG, Accelerometry (Dominant tremor frequency, occurrence rate, HOR)	Upper limbs and lower limbs rest, Postural and weight-holding tremor	i-iii) NR
(Zhao et al. 2020); China	75%	Comparative cross-sectional observation	50 (22 MSA-P & 28 MSA-C)	59.3 (NR)	26 M, 24 F	F-FDG-PET, MRI, H-Y stage, UPDRS	Rest and postural tremor	i ) L-Dopa / 375–750 ii) Yes (24% ) iii) No
(Calikusu et al. 2023); Turkey	88%	Retrospective descriptive study	19 (11 MSA-P & 8 MSA-C	59.9 (NR)	10 M, 9 F	MRI, Genetic analysis, EMG (in selected cases)	Rest tremor and myoclonus	i-iii) NR
(Pradhan and Tandon 2023); India	88%	Comparative cross-sectional observation	57 (39 MSA-P & 18 MSA-C)	60.1 (NR)	42 M, 15 F	MRI, Blood tests, UPDRS	Any tremor and pill-rolling	i) L-Dopa / NR ii) Yes (86%: 92.3% MSA-P, 72.2% MSA-C) iii) Yes (64%)
(Miki et al. 2019); UK	100%	Retrospective observational analysis	160 (103 MSA-P & 57 MSA-C)	56.7 AO; 63.7 AD (NR)	86 M, 74 F	Neuropathological Diagnosis	Rest, postural/action, Intention, jerky tremor, myoclonic postural/action, Polyminimyoclonus	i) L-Dopa / NR ii) Yes (40.5% moderate to good) iii) NR
(Xie et al. 2015); USA	100%	Descriptive cross-sectional	13 (7 MSA-P & 6 MSA-C)	66.4 at onset (NR)	8 M, 5 F	MRI	Rest, postural and action tremor	i) L-Dopa / 200-1300 ii) Yes (7/13 patients) iii) Yes (2–10 years)
(Gasca-Salas et al. 2013); Spain	90%	Retrospective observational analysis	3 MSA-P	67 (NR)	3 F	EMG	Upper limb orthostatic myoclonus	i) Piracetam / 1500 ii) Yes (mild) iii) NR
(Rusz et al. 2015); Czech Republic	100%	Descriptive cross-sectional	13 (10 MSA-P & 3 MSA-C)	60.8 (NR)	6 M, 7 F	FTRI	Vocal tremor	i-iii) NR
(Hlavnička et al. 2020); Czech Republic	90%	Descriptive cross-sectional study	20 (15MSA-P & 5 MSA-C)	63 (NR)	10 M, 10 F	EMG	Vocal tremor	i-iii) NR
(Li et al. 2018); China	100%	Descriptive cross-sectional study	69 (29 MSA-P & 40 MSA-C)	61.2 (NR)	40 M, 29 F	Posturography, EMG	Lower limb 3-Hz postural tremor	i-iii) NR
(Li et al. 2021); China	100%	Descriptive cross-sectional study	58 MSA-C	56.8 (NR)	37 M, 21 F	Posturography, EMG	Lower limb 3-Hz postural tremor	i-iii) NR
(Low et al. 2015); USA	100%	Cohort study	175 (126 MSA-P & 49 MSA-C)	63.4 AO (NR)	105 M, 70 F	UMSARSI + II, IV	Rest and postural tremor	i) L-Dopa / NR ii) Acute response: Yes (51.6%) iii) Sustained response: Yes (mean 3.2 years)
(Wang et al. 2025); China	100%	Descriptive cross-sectional study	133 (46P & 87 MSA-C	58.5 (NR)	85 M, 48 F	Posturography, EMG	Lower limb 3-Hz orthostatic tremor	i-iii) NR
**Interventional / trial studies**								
(Sinai et al. 2024); Israel	50%	Trial	5 MSA-P	NR (67–74)	2 M, 3 F	Median hemi-CRST, UMSARS III	Any upper extremity tremor	Median scores at hemi-CRST following VIM FUS: Baseline: 13 (*n* = 5, range 5–17 ) Immediately: 0 (*n* = 5, range 0–0), 1 Month: 0 (*n* = 5,, range 0–14) 6 Months: 3.5 (*n* = 4, range 0–10) 12 months: 1(*n* = 3, range 1–4)
(Jeong et al. 2017); South Korea	100%	Trial	49 MSA-C	62 (N/A)	30 M, 19 F	F-18 FP-CIT PET	Rest tremor	i) Dopaminergic medication / NR ii) Yes (7/22 patients) iii) NR
(Ye et al. 2024); Germany	64%	Trial	26 (15 MSA-P & 11 MSA-C)	62.5 (NR)	8 M, 18 F	MDS-UPDRS III, UMSARS I & II	Rest tremor & action tremor	Acute L-Dopa challenge: No improvement in 76.9%
